# Mind at rest, mind at risk: A prospective population-based study of sleep and subsequent mental disorders

**DOI:** 10.1016/j.sleepx.2025.100138

**Published:** 2025-01-16

**Authors:** Mari Hysing, Allison G. Harvey, Ann Kristin Skrindo Knudsen, Jens C. Skogen, Anne Reneflot, Børge Sivertsen

**Affiliations:** a Department of Psychosocial Science, University of Bergen, Bergen, Norway; b Department of Psychology, University of California, Berkeley, CA, USA; c Department of Disease Burden, Norwegian Institute of Public Health, Bergen, Norway; d Department of Health Promotion, Norwegian Institute of Public Health, Bergen, Norway; e Center for Alcohol & Drug Research, Stavanger University Hospital, Stavanger, Norway; f Centre for Evaluation of Public Health Measures, Norwegian Institute of Public Health, Oslo, Norway; g Department of Mental Health and Suicide, Norwegian Institute of Public Health, Oslo, Norway; h Department of Research & Innovation, Helse-Fonna HF, Haugesund, Norway

## Abstract

**Background:**

Depression and anxiety disorders are highly prevalent among young adults, with evidence suggesting sleep problems as key risk factors.

**Objective:**

This study aimed to examine the association between insomnia and sleep characteristics with major depressive episode (MDE) and anxiety disorders, and the association after accounting for baseline mental health symptoms.

**Methods:**

We conducted a prospective cohort study using data from the Students’ Health and Wellbeing Study (SHoT), surveying Norwegian higher education students aged 18 to 35 (N = 53,362). A diagnostic assessment of 10,460 participants was conducted in 2023. Self-reported insomnia, sleep duration, sleep onset latency, and wake after sleep onset were recorded in 2022. MDE and five types of anxiety disorders were assessed after one year using a self-administered CIDI 5.0. Analyses adjusted for age, sex, baseline mental health symptoms, and somatic conditions.

**Results:**

Insomnia in young adults was associated with a significantly increased risk of MDE (adjusted RR = 3.50, 95 % CI = 3.18–3.84) and generalized anxiety disorder (GAD) (adjusted RR = 2.82, 95 % CI = 2.55–3.12) one year later. Sleep duration showed a reversed J-shaped association with mental disorders, with both short and, to a lesser extent, long sleep durations linked to elevated risks, even after adjusting for baseline mental health symptoms and somatic conditions. Although the associations were attenuated after adjustment, they remained statistically significant.

**Conclusion:**

Sleep disturbances, including insomnia and abnormal sleep durations, predict mental health issues in young adults, even after accounting for baseline mental health and somatic health. Addressing sleep problems early may help prevent subsequent mental health conditions in this population.

## Highlights

• Insomnia, short and long sleep duration, long sleep onset latency and wake after sleep onset were associated with depression and anxiety disorders during the 1-year follow-up among young adult students.
• The strength of the associations exhibited a diagnosis-specific pattern, with the strongest associations observed between sleep problems and the prevalence of major depressive episode and generalized anxiety disorder.
• Insomnia, short and long sleep duration, long sleep onset latency and wake after sleep onset were associated with depression and anxiety There was a diagnosis-specific pattern, with the strongest associations between sleep problems and MDE and GAD.

## 1 Introduction

There has been a notable increase in the prevalence of anxiety and depression over the last decade, especially among young adults [1,2]. This concerning trend is accompanied by a parallel rise in sleep problems [3]. This has further strengthened the interest in sleep problems as a predictor of the development of depression and anxiety disorders. While it is widely acknowledged that a diverse spectrum of sleep indicators frequently co-occurs with depression and anxiety [[4], [5], [6]], a detailed understanding of how various facets of sleep may be related to subsequent mental disorders in young adulthood remains limited.

Convergent evidence suggests that sleep is a candidate causal mechanism that contributes to the development of mental health problems [7,8]. Specifically, a comprehensive meta-analysis involving adults disclosed a two-fold increased odds of developing depression among individuals with insomnia [8]. A meta-analysis of longitudinal studies of adolescents and young adults with insomnia unveiled a similar increased likelihood of developing a mood disorder [7]. In a seminal study of young adults, insomnia increased the risk of depression four-fold after three years [9]. Furthermore, in a longitudinal study of young women, sleep problems predicted the onset of a self-reported psychiatric diagnosis, encompassing both anxiety and depression [10].

The existing literature has some specific limitations. In most of the studies, the focus has been on insomnia as the main exposure, with depression serving as the principal outcome [4]. While a subset of studies have shown sleep problems to be a transdiagnostic risk factor for the onset of both depression and anxiety [11], other studies have indicated more diagnosis-specific relationships. For instance, in a longitudinal study involving youth, insomnia was identified as a predictor of depression onset, but not for anxiety disorder onset [12]. Furthermore, the majority of studies investigating the interplay between sleep and anxiety or depression have relied on screening questionnaires (e.g Refs. [10,13]), while others have employed broader diagnostic categories, such as anxiety disorders in general [9], without assessing potential different profiles across anxiety disorders. Among the studies that have included diagnostic outcomes, most of them are relatively small (e.g. Refs. 9,4) While insomnia has been extensively examined as a risk factor for later mental health problems, measures of sleep duration have received comparatively less scrutiny. This discrepancy was exemplified in a recent meta-analysis on short sleep duration and health in prospective studies, which identified an insufficient number of studies to facilitate a meta-analysis [14]. Only one of the two identified studies were on young adults, and it showed that men in medical school did not have a significant increased risk of later clinical depression during the follow up-period [15]. Hence, there is a pressing need to conduct a detailed exploration of this complex pattern of associations, encompassing both specific diagnostic categories and a wider range of sleep characteristics. Sleep duration and insomnia differ by gender [16], and there are also gender differences in prevalence of MDE and anxiety disorders, with a higher rate among women [17]. Further, sleep problems, and incidence rates of MDE anxiety may also differ across early adulthood [3,18], Thus, both sex and age need to be considered and included as control variables when assessing the association between sleep and MDE.

The aim of the present study was to 1) Assess the association between an extensive array of sleep characteristics extensive array of sleep characteristics—including insomnia, sleep duration, and the prevalence of depression and anxiety disorders in young adults during a 12-month follow-up and 2) to assess the association between sleep and depression and anxiety disorders after accounting for baseline mental health symptoms.

## 2 Methods

### 2.1 Setting and participants

The current study's primary population is drawn from the SHOT study (Students' Health and Wellbeing Study), survey focusing on Norwegian students enrolled in higher education. The SHOT2022 survey comprehensively explored various aspects of health and lifestyle and detailed information about SHOT has been published elsewhere [19].

During the survey period, SHOT2022 was distributed electronically through a web-based platform and was open for responses from February 8 to April 19. All full-time Norwegian students pursuing higher education, both domestically and abroad, were invited to participate. Efforts were made to raise awareness about the study through email, SMS, and information campaigns by numerous welfare organizations and educational institutions. The total number of students meeting the inclusion criteria for the study was 169,572 (58.4 % females), out of which 59,544 students completed the online questionnaires after receiving two reminders. This resulted in a response rate of 35.1 %. The response rates across the four health regions in Norway were relatively consistent, ranging from 32.1 % to 37.5 %, as determined from aggregated data obtained from the Norwegian State Educational Loan Fund.

When consenting to participate in the SHOT2022, students were given the option to express their interest in participating in a follow-up study on mental disorders. Detailed information about the participation flow in a CIDI study has been published elsewhere [20]. In short, 16,418 students were invited of the 53,362 participants aged between 18 and 35 years of whom 10,460 provided valid responses on at least one of the CIDI diagnostic sections, resulting in a conditional response rate of 63.7 %. The CIDI study took place between January 24 and February 6, 2023, approximately 12 months after the SHOT2022 survey was conducted.

### 2.2 Instruments

#### 2.2.1 Sleep duration and insomnia

Participants provided self-reported information regarding their typical bedtime and rise time, specified in hours and minutes separately for weekdays and weekends. From these data, the time in bed (TIB) was calculated as the duration between bedtime and rise time. Additionally, sleep onset latency (SOL) and wake after sleep onset (WASO) were recorded separately for weekdays and weekends, representing the time taken to fall asleep and the time spent awake after initially falling asleep, respectively. To determine sleep duration, the time spent on SOL and WASO was subtracted from TIB. Participants also reported the average number of nights per week they experienced difficulties initiating sleep (DIS), difficulties maintaining sleep (DMS), and early morning awakenings (EMA), as well as their level of daytime sleepiness and tiredness. Those experiencing sleep problems were asked about the duration of these problems. Insomnia disorder was operationalized based on three criteria, in accordance with DSM-5 criteria: 1) the presence of either DIS, DMS, or EMA for at least 3 nights per week, 2) experiencing daytime sleepiness and tiredness for at least 3 days per week, and 3) a duration of the sleep problems lasting at least 3 months. More detailed information about the sleep inventory used in SHoT study can be found elsewhere [3].

#### 2.2.2 Mental disorders: the CIDI

A newly developed self-administered electronic version of the World Health Organization (WHO) developed for the WHO World Mental Health (WMH) Surveys [21], was used for the data-collection [22]. A detailed description of the development and prevalence results from this self-administered version CIDI version has been published elsewhere [20]. In short, CIDI 5.0 is a standardized interview assessing 30-days, 12 months and lifetime prevalence for several mental and substance use disorders according to diagnostic criteria in the Diagnostic and Statistical Manual of Mental Disorders 5th edition (DSM-5) [23].

Subjects with a current mental disorder were defined as those who reported the presence of a mental disorder within the 30 days prior to the study. We also calculated 12-months and lifetime prevalence of mental disorders, and participants fulfilling either of these criteria, but NOT current mental disorder, were omitted from the statistical analyses, given the presents study's focus on mental disorders being present after the SHOT2022 data collection. The following mental disorders were included in the study: MDE, generalized anxiety disorder (GAD), panic disorder, specific phobia, agoraphobia, and social anxiety disorder. Operationalization of diagnoses was based on algorithms developed for CIDI 5.0 in the WMH Surveys Initiative.

#### 2.2.3 Sociodemographic information

##### 2.2.3.1 Time 1 covariates

Participants' age and sex information was derived from their 11-digit Norwegian national identity numbers. Background information was drawn from the SHOT2022 survey, participants were asked about their relationship status and whether they or their parents were born outside Norway.

Mental health problems in 2022 were assessed using the Hopkins Symptoms Checklist (HSCL-25) [24] which is a screening tool designed to detect symptoms of anxiety and depression. It is composed of a 10-item subscale for anxiety and a 15-item subscale for depression, with each item scored on a 4-point scale ranging from “not at all” (1) to “extremely” (4). The period of reference is the prior two weeks. An investigation of the factor structure based on the SHoT2014 dataset showed that a unidimensional model has optimal psychometric properties for application to student populations [25]. High scorers on the HSCL-25 were defined using recently validated cut-off values identified with the CIDI 5.0 as the reference standard: 1.96 for males and 2.20 for females [26].

##### 2.2.3.2 Time 2 covariates

Delayed Sleep Wake Phase Disorder: To establish a proxy for assessing DSWPD in line with the third and current edition of the International Classification of Sleep Disorders (American Academy of Sleep Medicine, 2005), we employed the following 5 criteria, as specified in Johnson et al. [27]1) minimum 1-h shift in sleep-onset AND wake times from the weekdays to the weekend, 2) complaint of frequent (≥3 days per week) difficulty falling asleep, 3) report of little or no (≤1 day per week) difficulty maintaining sleep, and 4) frequent difficulty awakening (oversleep “sometimes” or more often), and 5) at least 7 h of sleep per night on the weekends. No information was available concerning the students' desired bed time or their inability to fall asleep at the desired time which, according to ICSD-3, would be required to fulfill the criteria for a clinical diagnosis of DSWPD.

Somatic Conditions/illnesses: Physical disorders/conditions were assessed by self-report using a pre-defined list adapted to fit this age-cohort. This was based on a similar operationalization used in previous large population-based studies (the HUNT studies [28]). The list included atopic conditions (allergy and intolerances, asthma, eczema), neurological conditions (cerebral palsy, epilepsy, migraine, multiple sclerosis, rheumatoid arthritis), psychosomatic conditions (chronic fatigue syndrome/myalgic encephalomyelitis [CFS/ME], fibromyalgia and irritable bowel), and other somatic conditions (cancer, type 1 diabetes, and heart disease).

Participants’ use of hypnotic medications was assessed with the following two items: “How often during the past four weeks have you used the following medications: (1) Prescription sleep medication, and (2) Non-prescription sleep medication?” Responses were recorded on a four-point scale: (1) “Daily,” (2) “Weekly, but not daily,” (3) “Less than weekly,” and (4) “Not used in the past 4 weeks.” This measure allowed us to capture the frequency of both prescription and over-the-counter sleep medication use among the participants.

### 2.3 Statistical analyses

**Fig. 1:**
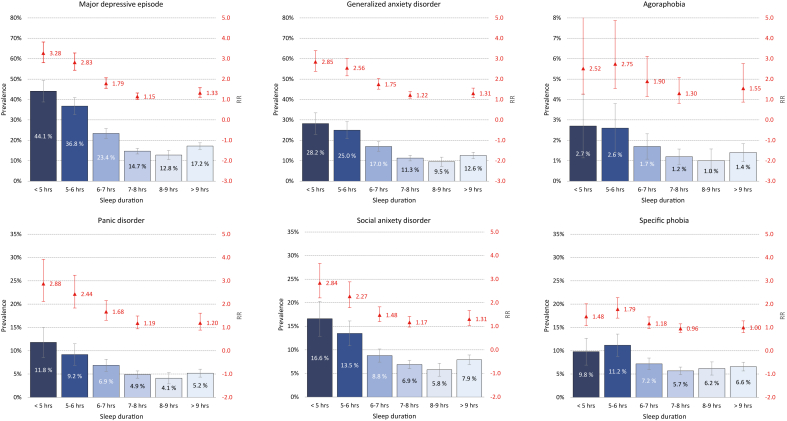
Association between sleep duration category in SHOT2022 and prevalence of mental disorder in 2023 (CIDI FU-study). Blue bars represent prevalence (left axis), while the red point estimates (right axis: on a natural scale) represent age-adjusted relative risk (RR). Error bars represent 95 % confidence intervals.

**Fig. 2:**
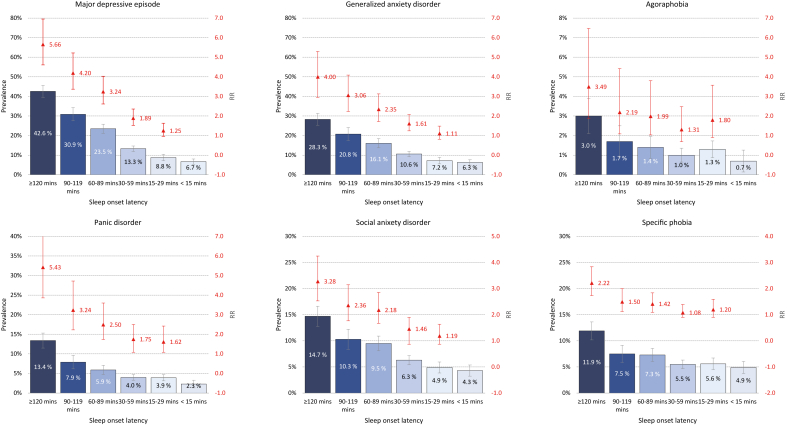
Association between sleep onset latency in SHOT2022 and prevalence of mental disorder in 2023 (CIDI FU-study). Blue bars represent prevalence (left axis), while the red point estimates (right axis: not on a logarithmic scale) represent age-adjusted relative risk (RR). Error bars represent 95 % confidence intervals.

**Fig. 3:**
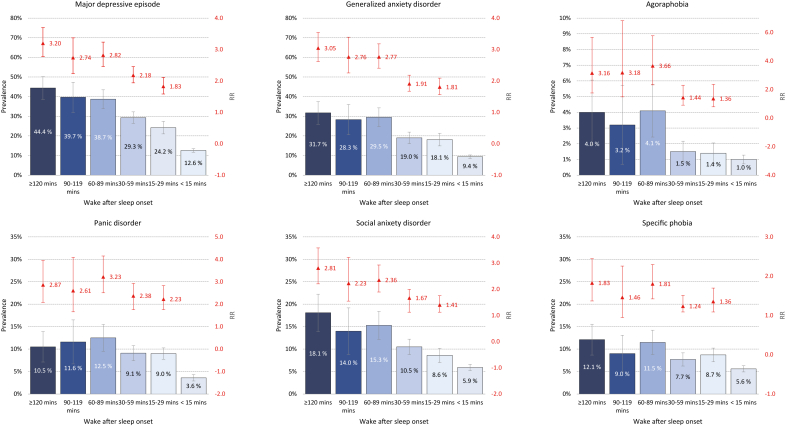
Association between wake after sleep onset in SHOT2022 and prevalence of mental disorder in 2023 (CIDI FU-study). Blue bars represent prevalence (left axis), while the red point estimates (right axis: not on a logarithmic scale) represent age-adjusted relative risk (RR). Error bars represent 95 % confidence intervals.

First, we calculated unweighted descriptive and sleep characteristics (age, sex, relationship status, country of birth, parental education, sleep duration and insomnia) of the responders and non-responders of the CIDI study, and the total SHOT2022 sample. Statistical comparisons using weighted data were made between CIDI responders vs. CIDI non-responders and CIDI responders vs. SHOT2022, using Chi-squared test (for categorical variables) or independent samples t-tests (for continuous variables). All analyses on the associations between sleep and mental disorders were weighted for sex, given the differences in sex distribution between the general student population (58.4 % females) and the current CIDI sample (70.6 % sample). Prevalence estimates for MDE and anxiety disorders, stratified by insomnia and sleep duration category, were then calculated. To further investigate the associations, we conducted log-link binomial regression analyses to calculate effect sizes for each mental disorder. Results are presented as risk ratios (RR) with 95 % confidence intervals. In our analysis, we implemented three models to account for different levels of confounding. Model 1 was adjusted for age and sex. In Model 2, we additionally adjusted for baseline symptoms of anxiety and depression, as measured by high scorers on the HSCL-25. Model 3 which adds DSWPD, use of hypnotic medication, and comorbid somatic illnesses, in addition to the variables from Model 2. The estimates shown in Fig. 1, Fig. 2, Fig. 3 are based on Model 1, while Models 1–3 are detailed in Supplementary Tables 1–3. All statistical analyses were performed using IBM SPSS version 28.

## 4 Results

### 4.1 Sample characteristics and representativeness

The characteristics of students who participated in both the CIDI study and the original SHOT2022 study are summarized in Table 1. Among the CIDI responders, the mean age was 24 years, with a predominance of females of Norwegian born versus foreign born, and approximately 50 % of them reported being single. The demographic features of the CIDI study participants were largely similar to those of the overall SHOT2022 study, except for a difference in sex distribution, with 70.6 % females in the CIDI sample compared to 66.4 % in SHOT2022. The analyses of students invited to participate in the CIDI study, but who did not respond, revealed no significant differences across demographic characteristics when compared to CIDI responders (see Table 2).

**Table 1:** Unweighted sociodemographic and sleep characteristics in 2022 of the CIDI responders, CIDI non-responders and the overall SHOT2022 sample.

Characteristic	CIDI responders (n = 10,460)	CIDI non-responders (n = 5958)	p-value^b^	SHOT2022^a^ (n = 53,362)	p-value^b^
Age, mean (SD)	24.03 (3.28)	23.97 (3.24)	.24	23.98 (1.85)	.14
Sex, % (n)			.97		<.00
Women	70.6 (7386)	70.4 (4196)		66.4 (35,423)	
Men	29.4 (3074)	29.6 (1762)		33.6 (17,939)	
Relationship status, % (n)			.16		.70
Single	51.2 (5359)	50.3 (2994)		51.0 (27,197)	
Boy-/girlfriend	22.4 (2343)	23.7 (1414)		22.8 (12,152)	
Cohabitant	22.7 (2375)	22.5 (1340)		22.6 (12,058)	
Married/registered partner	3.3 (345)	3.0 (178)		3.1 (1667)	
*Missing*	*0.4 (38)*	*0.5* (32)		*0.5 (288)*	
Self and/or parent(s) born abroad, % (n)			.17		.34
Born in Norway	81.2 (8491)	80.4 (4792)		80.1 (43,052)	
Born outside Norway	10.0 (1043)	10.9 (651)		10.4 (5541)	
*Missing*	*8.9 (926)*	*8.6* (515)		*8.9 (4769)*	
Sleep duration, Mean (SD) ^$^	7:29 (1:23)	7:26 (1:26)	.03	7:30 (1:22)	.45
*Missing, % (n)*	*3.5 (363)*	*4.7 (311)*			
Insomnia, % (n)	36.3 % (3778)	36.3 % (2381)	.457	33.9 % (18042)	<.001

SHOT2022: Students' Health and Wellbeing Study 2022; CIDI: Composite International Diagnostic Interview.

a Grand mean for the SHOT2022 sample aged 18–35.

b Compared with the CIDI responders group (p-values based on Chi-squared test (categorical variables) or *t*-test (continuous variables).

**Table 2:** Association between insomnia in SHOT2022 and prevalence of mental disorders in 2023 (the CIDI follow-up study).

	No insomnia		Insomnia			
Mental disorder	%^a^	(n)	%^a^	(n)	Adjusted relative risk^b^	(95 % CI)
No mental disorder	75.8 %	(3976)	42.2 %	(1170)		
Any mental disorder	24.2 %	(1266)	57.8 %	(1602)	2.10	(1.99–2.22)
Major depressive episode (n = 1566)	9.8 %	(522)	37.2 %	(964)	3.50	(3.18–3.84)
Generalized anxiety disorder (n = 1395)	7.9 %	(496)	24.9 %	(802)	2.82	(2.55–3.12)
Agoraphobia (n = 159)	.8 %	(55)	2.5 %	(88)	2.61	(1.89–3.58)
Panic disorder (n = 593)	3.3 %	(208)	10.2 %	(323)	2.66	(2.26–3.11)
Social anxiety disorder (n = 838)	5.2 %	(342)	12.9 %	(436)	2.23	(1.95–2.53)
Specific phobia (n = 765)	4.8 %	(318)	10.5 %	(367)	1.88	(1.63–2.14)

a Prevalence of mental disorders are weighted for sex.

b Adjusted for age and sex.

Regarding sleep duration, the average hours of sleep among students who completed the CIDI follow-up study (7:29, SD = 1:23) was slightly longer compared to students who were invited but did not respond (7:26, SD = 1:26; p = .03). There was no significant difference in sleep duration between the CIDI responders and the overall SHOT2022 sample. In terms of insomnia, CIDI responders had slightly, but significantly, higher insomnia prevalence (36.3 %) compared to the overall SHOT2022 sample (33.9 %), but there was no difference in insomnia prevalence between CIDI responders and non-responders (see Table 1).

### 4.2 Insomnia and prevalence of mental disorders

The prevalence of a MDE in 2023 among students with insomnia in 2022 was 37.2 %, compared to 9.8 % among students with no insomnia. As such, students with insomnia had a 3.5-fold increased risk (adjusted RR = 3.50, 95 % CI = 3.18–3.84) of MDE during the 1-year follow-up. Similar associations were also found between insomnia and anxiety disorders, but with lower effect sizes, ranging from specific phobia (RR = 1.88, 95 % CI = 1.63–2.14) to generalized anxiety disorder (RR = 2.82, 95 % CI = 2.55–3.12).

### 4.3 Sleep duration and prevalence of mental disorders

The associations between sleep duration and prevalence of subsequent mental disorders are detailed in Fig. 2. The results across most mental disorders and sleep duration revealed an ᒐ-shaped (reversed J-shaped) pattern. For example, compared to sleeping 8–9 h, students sleeping less than 5 h had a 3.28-fold increased risk of subsequent MDE, while the corresponding RRs for students sleeping 5–6 h and 6–7 h h, were RR = 2.83 and 1.79, respectively. A sleep duration of more than 9 h was also associated with increased risk (RR = 1.33).

Similar patterns, but with slightly lower risk estimates, were observed for both generalized anxiety disorder, panic disorder and social anxiety disorder (see Fig. 1 for details). And while short sleep was also significantly associated with agoraphobia and specific phobia, these patterns were less evident.

### 4.4 Sleep onset latency and prevalence of mental disorders

Fig. 2 illustrates the association between sleep onset latency (SOL) and the prevalence of subsequent mental disorders. The findings closely mirrored those of sleep duration, but with a more distinct dose-response relationship, the longer the SOL, the higher the risk of having a mental disorder during the 1-year follow-up. For instance, students taking over 2 h to fall asleep faced a 5.66-fold increased risk of experiencing a MDE compared to students who fell asleep within 15 min. For students with a SOL of 90–119 min and 60–89 min, the corresponding relative risks were 4.20 and 3.24, respectively. A similar pattern was observed for panic disorder, with dose-response relationships evident for generalized anxiety disorder (GAD) and social anxiety disorder, though the effect sizes were slightly smaller. Regarding agoraphobia and specific phobia, the association between SOL and these disorders was less evident, which was comparable to the findings for sleep duration.

### 4.5 Wake after sleep onset and prevalence of mental disorders

The associations between nocturnal wake time and prevalence of mental disorders are shown in Fig. 3. Like the graded relationship observed for SOL, WASO also showed a dose-response association with a MDE. However, the effect sizes for WASO were generally smaller compared to those observed for SOL. And although long WASO was significantly associated with all disorders, the graded associations were not as evident for agoraphobia, panic disorder, and specific phobia.

### 4.6 Sleep and mental disorders: adjusting for baseline symptoms of anxiety and depression, and health related factors

Supplementary Tables 1-3,

In Model 2, which adjusts for baseline anxiety and depression symptoms, risk estimates were notably reduced but still significant. For instance, the risk for major depressive episodes (MDE) associated with less than 5 h of sleep dropped from a relative risk (RR) of 5.68 (95 % CI: 4.63–6.98) in Model 1 to 2.69 (95 % CI: 2.21–3.28) in Model 2. Similarly, the risk for generalized anxiety disorder (GAD) fell from 4.01 (95 % CI: 3.28–4.90) to 1.97 (95 % CI: 1.62–2.39),

In Model 3, which further adjusts for DSWPD, hypnotic medication use, and comorbid somatic illnesses, the risk estimates decreased further. For example, the risk of MDE for less than 5 h of sleep fell to 2.38 (95 % CI: 1.93–2.93), and the risk for GAD dropped to 1.88 (95 % CI: 1.53–2.32).

## 5 Discussion

The present large-scale study reports on a unique link between a national study of student health including sleep from 2022, and a validated psychiatric diagnostic survey (CIDI 5.0) administered one year later. Both insomnia and sleep duration emerged as significant predictors of depression and anxiety The strengths of the associations varied across specific diagnostic categories, with the largest effect-sizes observed for MDE and GAD.

The results confirm insomnia as a significant risk factor for depression [7,8]. Moreover, the outcomes extend our knowledge in two key domains. First these findings highlight that insomnia also predicts anxiety disorders during a 1-year follow-up, aligning with previous investigations [10,11], yet contrasting with results from other research [12]. While insomnia can be seen as a transdiagnostic risk marker for anxiety, the present study underscores that this association is particularly pronounced in the context of GAD, and to a somewhat lesser extent, for other anxiety disorders, such as panic disorder and specific phobias. Second, the current findings also broaden our knowledge regarding which type of sleep problems are associated with mental disorders. Beyond insomnia, our investigation also found a significant association between sleep duration and mental disorders, characterized by an ᒐ-shaped association: both short, and to some extent long sleep duration predicted subsequent mental disorders, which also has been found in previous reports [29]. The short sleep duration was both a result of long sleep onset latency, and to a lesser degree nocturnal wake time.

A wide spectrum of mechanisms may potentially account for the role of sleep and depression and anxiety disorders, encompassing both common transdiagnostic pathways, and others more specific to the development of the distinct disorders. One potential common underlying factor may be worry, rumination and/or hyperarousal, which may contribute to both sleep problems and manifestation of depression and anxiety.[30 31], and which may be more related to MDE and GAD, than the other anxiety disorder which may be more context dependent. Sleep problems may also directly impact mood. For instance, short sleep duration has both been linked to reduced mood [32], and also to more negative affect the next day [33]. This cumulative effect may contribute to the both maintenance and development of depressive symptoms over time [34]. Similarly, acute sleep deprivation has been associated with heightened levels of state anxiety [35]. Further, poor sleep may compromise our capacity to cope with daily life stressors [36]. Finally, genetic influences may also play an important role in the association between insomnia and mental disorders over time [37]. To further unravel the intricate interplay and relative importance of these mechanisms, it is imperative that future studies be conducted with the aim of clarifying the causal pathways through which sleep ultimately contributes to the maintenance and development of depression and anxiety.

We have accounted for confounding variables by weighting for sex differences and adjusting for age and sex in the analysis. Additional adjustment for baseline symptoms of anxiety and depression a led to a substantial reduction in risk estimates for the associations between sleep parameters (SOL, WASO, and sleep duration) and subsequent mental disorders. Importantly, however the associations remained statistically significant, underscoring the independent link between poor sleep and later mental health outcomes. In a final model we also adjusted for somatic illness, DSWPD and medication which reduced the risk estimates further. Despite these reductions, the associations remained statistically significant, highlighting that even after accounting for these additional factors, poor sleep is still prospectively linked to a higher risk of mental disorders. These final analyses should be interpreted with caution, since there is a risk of overadjustment since these factors may partly be on the mechanistic pathway. Still, it supports the robustness of the association.

The current results underscore the importance of early identification and treatment of sleep problems. Treating sleep problems have shown to have a moderate effect on mental health problems in the general population [38]In the context of university students, interventions targeting sleep have shown a more moderate influence on improving sleep problems, and only small effects on improving symptoms of anxiety and depression [39]. With a finer focus, interventions aimed at extending sleep duration among young adults have shown to be effective [40,41]. Still, for young people with established and more severe sleep problems, cognitive and behavioral treatments are the most effective intervention [42].

Some methodological considerations warrant attention. A significant strength of this study is its prospective design, as the CIDI was administered 12 months after the initial data collection in 2022. However, it's important to underscore that the study design could not determine causality. An important strength is the utilization of diagnostic data acquired via a standardized and validated psychiatric survey, specifically the CIDI. Still, it should be mentioned that this approach diverged from traditional face-to-face interview by adopting a novel self-administered electronic questionnaire version of the CIDI 5.0, which poses some new challenges. While prior research employing CIDI 5.0 has shown no discrepancies in prevalence estimates between face-to-face and telephone interviews [43], the validation of this self-administered CIDI version against the conventional face-to-face CIDI remains unexplored. Another limitation regarding the SHOT2022 study pertains to our lack of knowledge regarding whether non-respondents of the SHOT2022 differ in their likelihood of having a mental disorder compared to respondents. Although existing evidence suggests that individuals who abstain from participating in health surveys often exhibit poorer health relative to those who do participate [44], it is worth considering that individuals might also be more inclined to partake in a survey if they perceive the subject matter to be personally relevant [45]. However, as previously reported [20], when comparing the level of mental health problems between participants and non-participants of the CIDI study, we found only marginal differences. Finally, we were also unable to examine the extent to which the current study sample accurately represents the reference population (i.e., Norwegian higher education students) in terms of demographic and educational characteristics. Still, it's worth noting that both the response rates and the HSCL-25 scores displayed notable similarity across all health regions in Norway, which all things considered may imply a reasonable level of representativeness in geographical terms. The use of a large, population-based design allowed us greater statistical power to detect associations, more generalizability, and allowed for the opportunity to reduce potential omitted variable bias, and is thus a significant strength of the current study.

In conclusion, this study underscores the significant link between sleep characteristics, including insomnia and sleep durations, and subsequent mental disorders among young adults. The study's findings advocate for the early identification and intervention of sleep disturbances as preventive measures for mental health problems.

## CRediT authorship contribution statement

**Mari Hysing:** Writing – review & editing, Writing – original draft, Methodology, Formal analysis, Conceptualization. **Allison G. Harvey:** Writing – review & editing. **Ann Kristin Skrindo Knudsen:** Writing – review & editing, Methodology. **Jens C. Skogen:** Writing – review & editing. **Anne Reneflot:** Writing – review & editing, Methodology. **Børge Sivertsen:** Writing – review & editing, Writing – original draft, Project administration, Methodology, Investigation, Formal analysis, Conceptualization.

## 3 Ethics

The study was approved by the Regional Committee for Medical and Health Research Ethics in Western Norway (no. 2022/326437).

## Data availability statement

The SHoT2022 dataset is administered by the National Institute of Public Health. Approval from a Norwegian regional committee for medical and health research ethics [https://helseforskning.etikkom.no] is a prerequisite.

## Funding

SHoT2022 was supported by the 10.13039/501100017488Norwegian Ministry of Education and Research and the 10.13039/501100003506Norwegian Ministry of Health and Care Services.

## Declaration of competing interest

The authors declare that they have no known competing financial interests or personal relationships that could have appeared to influence the work reported in this paper.
