# Supplementary material for: Mind at rest, mind at risk: A prospective population-based study of sleep and subsequent mental disorders
Source: Sleep Med X. 2025 Jan 16;9:100138. doi: 10.1016/j.sleepx.2025.100138 (PMC11791349; doi:10.1016/j.sleepx.2025.100138)
Supplement: Multimedia component 1 [file mmc1.docx]

| **Supplementary Table 1. Relationship Between Sleep Onset Latency in SHOT 2022 and Risk of Mental Disorders After One Year, Adjusted for Demographic and Clinical Covariates Across Three Models.** | | | | | | | | | | |
| --- | --- | --- | --- | --- | --- | --- | --- | --- | --- | --- |
| **Sleeo onset latency** |  | **Model 1** |  |  | **Model 2** |  |  | **Model 3** |  |  |
|  |  | RR | 95% CI- | 95% CI+ | RR | 95% CI- | 95% CI+ | RR | 95% CI- | 95% CI+ |
| Major depressive episode | ≥120 mins | 5.68 | 4.63 | 6.98 | 2.69 | 2.21 | 3.28 | 2.38 | 1.93 | 2.93 |
|  | 90-119 mins | 4.20 | 3.37 | 5.23 | 2.31 | 1.87 | 2.84 | 2.10 | 1.69 | 2.60 |
|  | 60-89 mins | 3.26 | 2.62 | 4.04 | 1.98 | 1.61 | 2.43 | 1.90 | 1.54 | 2.35 |
|  | 30-59 mins | 1.90 | 1.52 | 2.36 | 1.46 | 1.19 | 1.80 | 1.43 | 1.15 | 1.77 |
|  | 15-29 mins | 1.26 | 0.96 | 1.64 | 1.17 | 0.91 | 1.50 | 1.16 | 0.89 | 1.50 |
|  | < 15 mins | 1.00 |  |  | 1.00 |  |  | 1.00 |  |  |
| Generalized anxiety disorder | ≥120 mins | 4.01 | 3.28 | 4.90 | 1.97 | 1.62 | 2.39 | 1.88 | 1.53 | 2.32 |
|  | 90-119 mins | 3.06 | 2.46 | 3.81 | 1.66 | 1.34 | 2.04 | 1.62 | 1.30 | 2.02 |
|  | 60-89 mins | 2.36 | 1.91 | 2.92 | 1.46 | 1.19 | 1.79 | 1.44 | 1.17 | 1.79 |
|  | 30-59 mins | 1.62 | 1.31 | 1.99 | 1.19 | 0.97 | 1.46 | 1.21 | 0.98 | 1.49 |
|  | 15-29 mins | 1.11 | 0.86 | 1.44 | 0.99 | 0.77 | 1.27 | 1.03 | 0.79 | 1.32 |
|  | < 15 mins | 1.00 |  |  | 1.00 |  |  | 1.00 |  |  |
| Agoraphobia | ≥120 mins | 3.51 | 1.90 | 6.51 | 1.92 | 1.03 | 3.58 | 1.82 | 0.94 | 3.52 |
|  | 90-119 mins | 2.20 | 1.09 | 4.44 | 1.33 | 0.66 | 2.69 | 1.26 | 0.60 | 2.64 |
|  | 60-89 mins | 2.00 | 1.04 | 3.83 | 1.36 | 0.71 | 2.61 | 1.24 | 0.62 | 2.46 |
|  | 30-59 mins | 1.31 | 0.69 | 2.49 | 1.04 | 0.55 | 1.97 | 1.02 | 0.52 | 1.99 |
|  | 15-29 mins | 1.88 | 0.95 | 3.72 | 1.75 | 0.89 | 3.45 | 1.90 | 0.95 | 3.81 |
|  | < 15 mins | 1.00 |  |  | 1.00 |  |  | 1.00 |  |  |
| Panic disorder | ≥120 mins | 5.43 | 3.87 | 7.64 | 3.18 | 2.26 | 4.48 | 2.78 | 1.93 | 3.99 |
|  | 90-119 mins | 3.24 | 2.23 | 4.72 | 2.05 | 1.41 | 2.98 | 1.87 | 1.26 | 2.77 |
|  | 60-89 mins | 2.50 | 1.74 | 3.60 | 1.82 | 1.26 | 2.61 | 1.80 | 1.24 | 2.63 |
|  | 30-59 mins | 1.76 | 1.23 | 2.51 | 1.42 | 1.00 | 2.02 | 1.44 | 1.00 | 2.08 |
|  | 15-29 mins | 1.61 | 1.08 | 2.42 | 1.51 | 1.01 | 2.25 | 1.60 | 1.06 | 2.42 |
|  | < 15 mins | 1.00 |  |  | 1.00 |  |  | 1.00 |  |  |
| Social anxiety disorder | ≥120 mins | 3.29 | 2.54 | 4.26 | 1.88 | 1.46 | 2.44 | 1.74 | 1.33 | 2.27 |
|  | 90-119 mins | 2.37 | 1.77 | 3.15 | 1.48 | 1.11 | 1.97 | 1.33 | 0.99 | 1.79 |
|  | 60-89 mins | 2.20 | 1.69 | 2.88 | 1.54 | 1.18 | 2.00 | 1.43 | 1.09 | 1.88 |
|  | 30-59 mins | 1.46 | 1.12 | 1.90 | 1.18 | 0.91 | 1.53 | 1.10 | 0.84 | 1.43 |
|  | 15-29 mins | 1.20 | 0.88 | 1.65 | 1.13 | 0.83 | 1.54 | 1.09 | 0.80 | 1.50 |
|  | < 15 mins | 1.00 |  |  | 1.00 |  |  | 1.00 |  |  |
| Specific phobia | ≥120 mins | 2.22 | 1.74 | 2.84 | 1.71 | 1.33 | 2.20 | 1.71 | 1.32 | 2.22 |
|  | 90-119 mins | 1.50 | 1.12 | 2.00 | 1.21 | 0.90 | 1.62 | 1.17 | 0.86 | 1.58 |
|  | 60-89 mins | 1.42 | 1.09 | 1.84 | 1.21 | 0.93 | 1.58 | 1.20 | 0.92 | 1.57 |
|  | 30-59 mins | 1.08 | 0.84 | 1.39 | 0.99 | 0.77 | 1.27 | 0.97 | 0.75 | 1.26 |
|  | 15-29 mins | 1.19 | 0.90 | 1.59 | 1.17 | 0.88 | 1.55 | 1.21 | 0.91 | 1.62 |
|  | < 15 mins | 1.00 |  |  | 1.00 |  |  | 1.00 |  |  |
| Model 1: Adjusted for aghe and sex; Model 2: Model 1 + Symptoms of anxiety and depression (HSCL-25 high scorers); Model 3: Model 2 + Delayed Sleep Phase Disorder, use of sleep medicaiton and comorbid somatic conditions/illnesses. | | | | | | | | | | |

| **Supplementary Table 2. Relationship Between Wake After Sleep Onset in SHOT 2022 and Risk of Mental Disorders After One Year, Adjusted for Demographic and Clinical Covariates Across Three Models.** | | | | | | | | | | |
| --- | --- | --- | --- | --- | --- | --- | --- | --- | --- | --- |
| **Wake after sleep onset** |  | **Model 1** |  |  | **Model 2** |  |  | **Model 3** |  |  |
|  |  | RR | 95% CI- | 95% CI+ | RR | 95% CI- | 95% CI+ | RR | 95% CI- | 95% CI+ |
| Major depressive episode | ≥120 mins | 3.20 | 2.77 | 3.70 | 1.72 | 1.51 | 1.96 | 1.51 | 1.32 | 1.72 |
|  | 90-119 mins | 2.76 | 2.24 | 3.39 | 1.81 | 1.52 | 2.16 | 1.66 | 1.41 | 1.96 |
|  | 60-89 mins | 2.82 | 2.46 | 3.24 | 1.64 | 1.45 | 1.86 | 1.49 | 1.32 | 1.69 |
|  | 30-59 mins | 2.19 | 1.95 | 2.46 | 1.50 | 1.35 | 1.67 | 1.40 | 1.26 | 1.56 |
|  | 15-29 mins | 1.84 | 1.60 | 2.13 | 1.41 | 1.24 | 1.60 | 1.33 | 1.17 | 1.52 |
|  | < 15 mins | 1.00 |  |  | 1.00 |  |  | 1.00 |  |  |
| Generalized anxiety disorder | ≥120 mins | 3.04 | 2.57 | 3.59 | 1.73 | 1.47 | 2.02 | 1.62 | 1.38 | 1.90 |
|  | 90-119 mins | 2.77 | 2.19 | 3.50 | 1.88 | 1.53 | 2.32 | 1.80 | 1.47 | 2.22 |
|  | 60-89 mins | 2.77 | 2.38 | 3.22 | 1.71 | 1.48 | 1.97 | 1.59 | 1.38 | 1.84 |
|  | 30-59 mins | 1.92 | 1.68 | 2.19 | 1.35 | 1.19 | 1.53 | 1.30 | 1.14 | 1.47 |
|  | 15-29 mins | 1.82 | 1.56 | 2.12 | 1.42 | 1.23 | 1.64 | 1.38 | 1.19 | 1.59 |
|  | < 15 mins | 1.00 |  |  | 1.00 |  |  | 1.00 |  |  |
| Agoraphobia | ≥120 mins | 3.14 | 1.75 | 5.63 | 1.99 | 1.11 | 3.58 | 1.57 | 0.81 | 3.05 |
|  | 90-119 mins | 3.18 | 1.48 | 6.82 | 2.36 | 1.10 | 5.03 | 2.35 | 1.09 | 5.04 |
|  | 60-89 mins | 3.67 | 2.33 | 5.77 | 2.46 | 1.56 | 3.88 | 2.41 | 1.50 | 3.87 |
|  | 30-59 mins | 1.44 | 0.91 | 2.30 | 1.07 | 0.67 | 1.71 | 1.07 | 0.66 | 1.73 |
|  | 15-29 mins | 1.46 | 0.85 | 2.49 | 1.18 | 0.69 | 2.02 | 1.23 | 0.71 | 2.11 |
|  | < 15 mins | 1.00 |  |  | 1.00 |  |  | 1.00 |  |  |
| Panic disorder | ≥120 mins | 2.87 | 2.08 | 3.95 | 1.89 | 1.38 | 2.61 | 1.67 | 1.20 | 2.33 |
|  | 90-119 mins | 2.62 | 1.67 | 4.11 | 2.00 | 1.28 | 3.11 | 1.82 | 1.17 | 2.82 |
|  | 60-89 mins | 3.23 | 2.52 | 4.16 | 2.27 | 1.77 | 2.92 | 2.13 | 1.66 | 2.74 |
|  | 30-59 mins | 2.39 | 1.94 | 2.93 | 1.83 | 1.49 | 2.25 | 1.71 | 1.38 | 2.10 |
|  | 15-29 mins | 2.23 | 1.76 | 2.84 | 1.84 | 1.46 | 2.33 | 1.80 | 1.42 | 2.28 |
|  | < 15 mins | 1.00 |  |  | 1.00 |  |  | 1.00 |  |  |
| Social anxiety disorder | ≥120 mins | 2.80 | 2.20 | 3.57 | 1.83 | 1.44 | 2.32 | 1.75 | 1.36 | 2.25 |
|  | 90-119 mins | 2.23 | 1.55 | 3.22 | 1.70 | 1.20 | 2.43 | 1.73 | 1.21 | 2.47 |
|  | 60-89 mins | 2.35 | 1.89 | 2.92 | 1.64 | 1.32 | 2.03 | 1.68 | 1.35 | 2.09 |
|  | 30-59 mins | 1.67 | 1.40 | 2.00 | 1.27 | 1.06 | 1.51 | 1.26 | 1.05 | 1.51 |
|  | 15-29 mins | 1.42 | 1.14 | 1.77 | 1.18 | 0.95 | 1.46 | 1.17 | 0.94 | 1.47 |
|  | < 15 mins | 1.00 |  |  | 1.00 |  |  | 1.00 |  |  |
| Specific phobia | ≥120 mins | 1.83 | 1.37 | 2.45 | 1.45 | 1.09 | 1.95 | 1.34 | 0.99 | 1.83 |
|  | 90-119 mins | 1.47 | 0.95 | 2.27 | 1.27 | 0.82 | 1.95 | 1.24 | 0.81 | 1.92 |
|  | 60-89 mins | 1.81 | 1.42 | 2.30 | 1.48 | 1.17 | 1.89 | 1.41 | 1.10 | 1.82 |
|  | 30-59 mins | 1.24 | 1.02 | 1.52 | 1.07 | 0.88 | 1.31 | 1.06 | 0.86 | 1.30 |
|  | 15-29 mins | 1.36 | 1.09 | 1.70 | 1.23 | 0.99 | 1.54 | 1.19 | 0.95 | 1.49 |
|  | < 15 mins | 1.00 |  | 0.00 | 1.00 |  |  | 1.00 |  | 0 |
| Model 1: Adjusted for aghe and sex; Model 2: Model 1 + Symptoms of anxiety and depression (HSCL-25 high scorers); Model 3: Model 2 + Delayed Sleep Phase Disorder, use of sleep medicaiton and comorbid somatic conditions/illnesses. | | | | | | | | | | |

| **Supplementary Table 3. Relationship Between Sleep Duration in SHOT 2022 and Risk of Mental Disorders After One Year, Adjusted for Demographic and Clinical Covariates Across Three Models..** | | | | | | | | | | |
| --- | --- | --- | --- | --- | --- | --- | --- | --- | --- | --- |
| **Sleep duration** |  | **Model 1** |  |  | **Model 2** |  |  | **Model 3** |  |  |
|  |  | RR | 95% CI- | 95% CI+ | RR | 95% CI- | 95% CI+ | RR | 95% CI- | 95% CI+ |
| Major depressive episode | < 5 hrs | 5.68 | 4.63 | 6.98 | 2.69 | 2.21 | 3.28 | 2.38 | 1.93 | 2.93 |
|  | 5-6 hrs | 4.20 | 3.37 | 5.23 | 2.31 | 1.87 | 2.84 | 2.10 | 1.69 | 2.60 |
|  | 6-7 hrs | 3.26 | 2.62 | 4.04 | 1.98 | 1.61 | 2.43 | 1.90 | 1.54 | 2.35 |
|  | 7-8 hrs | 1.90 | 1.52 | 2.36 | 1.46 | 1.19 | 1.80 | 1.43 | 1.15 | 1.77 |
|  | > 9 hrs | 1.26 | 0.96 | 1.64 | 1.17 | 0.91 | 1.50 | 1.16 | 0.89 | 1.50 |
|  | 8-9 hrs | 1.00 |  |  | 1.00 |  |  | 1.00 |  |  |
| Generalized anxiety disorder | < 5 hrs | 4.01 | 3.28 | 4.90 | 1.97 | 1.62 | 2.39 | 1.88 | 1.53 | 2.32 |
|  | 5-6 hrs | 3.06 | 2.46 | 3.81 | 1.66 | 1.34 | 2.04 | 1.62 | 1.30 | 2.02 |
|  | 6-7 hrs | 2.36 | 1.91 | 2.92 | 1.46 | 1.19 | 1.79 | 1.44 | 1.17 | 1.79 |
|  | 7-8 hrs | 1.62 | 1.31 | 1.99 | 1.19 | 0.97 | 1.46 | 1.21 | 0.98 | 1.49 |
|  | > 9 hrs | 1.11 | 0.86 | 1.44 | 0.99 | 0.77 | 1.27 | 1.03 | 0.79 | 1.32 |
|  | 8-9 hrs | 1.00 |  |  | 1.00 |  |  | 1.00 |  |  |
| Agoraphobia | < 5 hrs | 3.51 | 1.90 | 6.51 | 1.92 | 1.03 | 3.58 | 1.82 | 0.94 | 3.52 |
|  | 5-6 hrs | 2.20 | 1.09 | 4.44 | 1.33 | 0.66 | 2.69 | 1.26 | 0.60 | 2.64 |
|  | 6-7 hrs | 2.00 | 1.04 | 3.83 | 1.36 | 0.71 | 2.61 | 1.24 | 0.62 | 2.46 |
|  | 7-8 hrs | 1.31 | 0.69 | 2.49 | 1.04 | 0.55 | 1.97 | 1.02 | 0.52 | 1.99 |
|  | > 9 hrs | 1.88 | 0.95 | 3.72 | 1.75 | 0.89 | 3.45 | 1.90 | 0.95 | 3.81 |
|  | 8-9 hrs | 1.00 |  |  | 1.00 |  |  | 1.00 |  |  |
| Panic disorder | < 5 hrs | 5.43 | 3.87 | 7.64 | 3.18 | 2.26 | 4.48 | 2.78 | 1.93 | 3.99 |
|  | 5-6 hrs | 3.24 | 2.23 | 4.72 | 2.05 | 1.41 | 2.98 | 1.87 | 1.26 | 2.77 |
|  | 6-7 hrs | 2.50 | 1.74 | 3.60 | 1.82 | 1.26 | 2.61 | 1.80 | 1.24 | 2.63 |
|  | 7-8 hrs | 1.76 | 1.23 | 2.51 | 1.42 | 1.00 | 2.02 | 1.44 | 1.00 | 2.08 |
|  | > 9 hrs | 1.61 | 1.08 | 2.42 | 1.51 | 1.01 | 2.25 | 1.60 | 1.06 | 2.42 |
|  | 8-9 hrs | 1.00 |  |  | 1.00 |  |  | 1.00 |  |  |
| Social anxiety disorder | < 5 hrs | 3.29 | 2.54 | 4.26 | 1.88 | 1.46 | 2.44 | 1.74 | 1.33 | 2.27 |
|  | 5-6 hrs | 2.37 | 1.77 | 3.15 | 1.48 | 1.11 | 1.97 | 1.33 | 0.99 | 1.79 |
|  | 6-7 hrs | 2.20 | 1.69 | 2.88 | 1.54 | 1.18 | 2.00 | 1.43 | 1.09 | 1.88 |
|  | 7-8 hrs | 1.46 | 1.12 | 1.90 | 1.18 | 0.91 | 1.53 | 1.10 | 0.84 | 1.43 |
|  | > 9 hrs | 1.20 | 0.88 | 1.65 | 1.13 | 0.83 | 1.54 | 1.09 | 0.80 | 1.50 |
|  | 8-9 hrs | 1.00 |  |  | 1.00 |  |  | 1.00 |  |  |
| Specific phobia | < 5 hrs | 2.22 | 1.74 | 2.84 | 1.71 | 1.33 | 2.20 | 1.71 | 1.32 | 2.22 |
|  | 5-6 hrs | 1.50 | 1.12 | 2.00 | 1.21 | 0.90 | 1.62 | 1.17 | 0.86 | 1.58 |
|  | 6-7 hrs | 1.42 | 1.09 | 1.84 | 1.21 | 0.93 | 1.58 | 1.20 | 0.92 | 1.57 |
|  | 7-8 hrs | 1.08 | 0.84 | 1.39 | 0.99 | 0.77 | 1.27 | 0.97 | 0.75 | 1.26 |
|  | > 9 hrs | 1.19 | 0.90 | 1.59 | 1.17 | 0.88 | 1.55 | 1.21 | 0.91 | 1.62 |
|  | 8-9 hrs | 1.00 |  |  | 1.00 |  |  | 1.00 |  |  |
| Model 1: Adjusted for aghe and sex; Model 2: Model 1 + Symptoms of anxiety and depression (HSCL-25 high scorers); Model 3: Model 2 + Delayed Sleep Phase Disorder, use of sleep medicaiton and comorbid somatic conditions/illnesses. | | | | | | | | | | |
